# Effects of Infill Line Multiplier and Patterns on Mechanical Properties of Lightweight and Resilient Hollow Section Products Manufactured Using Fused Filament Fabrication

**DOI:** 10.3390/polym15122585

**Published:** 2023-06-06

**Authors:** Jibran Khaliq, Dharma Raj Gurrapu, Farah Elfakhri

**Affiliations:** Department of Mechanical and Construction Engineering, Faculty of Engineering and Environment, Northumbria University, Newcastle Upon Tyne NE1 8ST, UK; dharma.gurrapu@northumbria.ac.uk (D.R.G.); farah.elfakhri@northumbria.ac.uk (F.E.)

**Keywords:** 3D printing, infill density, infill pattern, PLA, infill line multiplier

## Abstract

Fused Filament Fabrication (FFF) is a popular additive manufacturing process for creating prototypes and end-use products. Infill patterns, which fill the interior of hollow FFF-printed objects, play a crucial role in determining the mechanical properties and structural integrity of hollow structures. This study investigates the effects of infill line multipliers and different infill patterns (hexagonal, grid, and triangle) on the mechanical properties of 3D printed hollow structures. Thermoplastic poly lactic acid (PLA) was used as the material for 3D-printed components. Infill densities of 25%, 50%, and 75% were chosen, along with a line multiplier of one. The results indicate that the hexagonal infill pattern consistently demonstrated the highest Ultimate Tensile Strength (UTS) of 1.86 MPa across all infill densities, out-performing the other two patterns. To maintain a sample weight below 10 g, a two-line multiplier was utilised for a 25% infill density sample. Remarkably, this combination exhibited a UTS value of 3.57 MPa, which is comparable to samples printed at 50% infill density, which were 3.83 MPa. This research highlights the importance of line multiplier in combination with infill density and infill pattens to ensuring the achievement of the desired mechanical properties in the final product.

## 1. Introduction

Fused Filament Fabrication (FFF) is a widely used additive manufacturing technique known for its ability to create lightweight and resilient hollow section products [1,2]. The 3D-printing processes provide a broad vision and scope for meeting the demand for environmentally responsible production to create the desired model or specimen with little waste, regardless of its shape [3,4]. FFF is a popular and promising 3D printing technique in which fused polymer is put down layer by layer on a platform to print components with complicated geometries and suitable mechanical qualities [5]. Using FFF, printed objects with tunable mechanical properties, including tensile strength, flexural strength, and impact resistance, can be fabricated. Polylactic Acid (PLA) gained popularity in 3D printing because of its excellent mechanical qualities, high dependability, low cost, dimensional precision, and environment friendly nature (being made from renewable biomass) [6,7]. Pure polymer products manufactured using FFF lack load-bearing qualities, while most 3D-printed polymer materials are currently unsuitable for use as structural components [8,9]. However, depending on the nature of the printing filament, FFF technology can print the polymer with reinforcement, whether continuous or discontinuous [10,11,12]; however, this method incurs additional costs and complicates the manufacturing process. Researchers investigated the effect of diverse mechanical responses, such as bending, tension, and torsion, on 3D-printed materials [8,13,14,15,16,17]. Although 3D printing is an automated technique, the resulting properties of the printed materials heavily depend upon the processing parameters used. It was reported that the infill density plays a critical role in determining the deformation and strength characteristics of the material, while keeping the costs down [18]. While 100% infill density provides the greatest strength, it can have a negative impact on total cost through increasing material consumption and printing times. Given the nature of the application, some 3D-printed objects may not require 100% infill density to fulfill their load-bearing applications. As a result, it is critical to find a tradeoff between infill density and the end-use of the product to enhance productivity for mass-scale manufacturing lines. The internal structure of 3D printed parts is critical to their mechanical properties. A higher infill density results in a more compact internal structure, which leads to a more even distribution of stress throughout the part [19]. This even distribution of stress results in a higher ultimate tensile strength and Young’s Modulus. In contrast, a lower infill density leads to a less compact internal structure, which results in a less even distribution of stress throughout the part, decreasing Young’s Modulus and Ultimate Tensile Strength (UTS) [20]. Reduced infill densities can lead to greater interstices in the structure, resulting in higher porosity, which may affect the strength of the component. Hence, understanding the best practices for the setup of “input parameters” is crucial since, in the open software chain, there are many available for setup. Several studies utilised laminate mechanics and classical laminate theory to characterise the material behavior of FFF synthesised parts [21]. The orientation of fibers in the layers, stacking order, and build orientation are key factors that influence the final behavior of 3D-printed parts. Since 3D printing is a layer-by-layer manufacturing technique that resembles a laminate structure. While laminate models can capture the effects of fiber orientation and stacking order, they are unable to account for the impact of build orientation on the material behavior of printed parts [22]. Various infill patterns, such as honeycomb, square, and zigzag, were investigated to quantify the stiffness and strength of lightweight cellular PLA parts under uniaxial tensile loading and flexural loading, both edgewise and flatwise [20,23,24,25]. It was observed that the infill patterns have less of an influence on mechanical properties compared to the infill density [26]. The same effect was observed in PLA, Polyether Ether Ketone- (PEEK), Acrylonitrile Butadiene Styrene—(ABS), and Polyethylene Terephthalate Glycol (PETG)-based materials [27]. A honeycomb structure was found to be the best energy absorbing infill pattern when tested under a quasi-static compression test [28]. However, Rodriguez et al. reported no effect of infill density and infill patterns on mechanical properties; rather, the angle of layer stacking was found to be the crucial factor [29].

The build orientation of FFF printed parts plays a crucial role in determining their mechanical properties. By examining the impact of factors such as layer thickness, print orientation, and shell thickness, researchers gained insights into how these parameters influence the mechanical behavior of FFF-printed parts [7,15,30,31,32]. Understanding the failure modes through detailed microstructural analysis provides valuable information for optimizing FFF printing processes and improving the overall reliability of printed parts [14,18].

Periodic cellular materials are often used to reduce the weight of components [9]. These structures often imitate patterns found in nature and have complex features that require advanced manufacturing techniques to fabricate. The mechanical properties of 3D-printed objects are affected by a number of printing parameters, including print temperature, build orientation, nozzle dimensions, extrusion rate, infill pattern/density, and layer thickness [7,14]. The line multiplier or extrusion width is an important parameter in FFF technology, affecting the mechanical properties of 3D printed samples. The line multiplier determines the width of the printed lines that constitute the infill and perimeter of the object. It also affects the bonding between the layers and the surface finish of the product. Several researchers investigated the crucial effect of processing parameters on mechanical properties, specifically focusing on enhancing UTS; however, finding a single set of parameters to enhance UTS proved to be challenging [18,33]. The meso- and macro-structures of 3D printed objects can be affected by changes in infill density. It is essential to note that increasing the infill density can also increase the printing time and material usage, which can increase the overall cost of production. Therefore, a balance should be achieved between the targeted mechanical properties and the manufacturing expenses. For certain applications, such as those in the aerospace or biomedical domains, the intended mechanical properties might carry greater significance than the production costs. In other applications, where the cost is a significant factor, a lower infill density may be used [5].

The line multiplier EM is a critical parameter in FFF that regulates filament feed to the nozzle; however, very few reports of this process exist. Ghobrani et al. investigated this effect by studying line multipliers 1, 1.1, and 1.2 in ABS-based samples with 100% infill density [34]. The EM controls the extruder gears’ rotational speed; however, factors such as filament viscosity, nozzle characteristics, and feeding mechanism affect the actual extrusion amount. Hence, to the best of our knowledge, there are no reports that investigated the combined effect of line multiplier on infill density and infill patterns at a constant weight. The primary objective of this study is to explore the correlation between a combination of processing parameters, such as infill line multiplier together with infill density and infill patterns for lightweight hollow structures, on the mechanical properties of 3D-printed PLA. Specifically, the study aims to understand the impact of infill density, infill pattern, and line multiplier on the mechanical properties of lightweight hollow structures. To achieve this goal, three different infill patterns (triangular, grid, and hexagonal) were utilised to create samples for analysis with one-line multipliers. Subsequently, samples with two-line multipliers that weighed less than 10 g were printed for a comparative analysis on mechanical properties. The mechanical properties of the printed structures were evaluated through quasi-static uniaxial tensile tests conducted on printed samples. The results will shed light on the significance of these parameters in optimising the 3D-printing process for PLA materials. The findings will contribute to advancing the understanding of PLA’s behaviour under different processing conditions, enabling the development of improved strategies for achieving desired mechanical properties in hollow 3D-printed structures.

## 2. Materials and Methods

**Figure 1:**
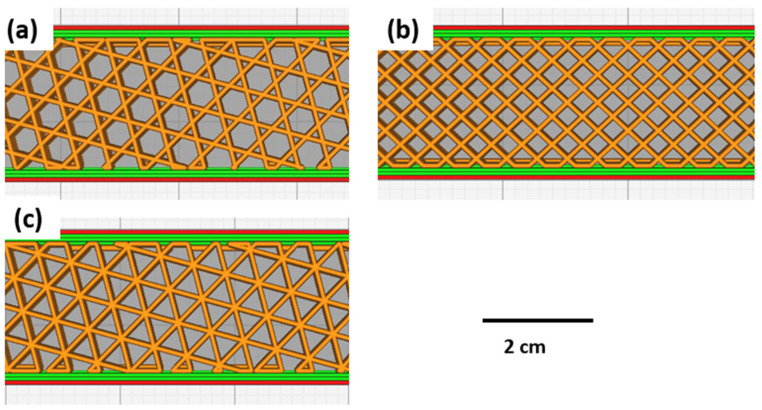
Infill patterns for 3D printed samples: (**a**) hexagonal pattern; (**b**) grid pattern; (**c**) triangular pattern.

To print the PLA samples, PLA filament (diameter of 1.75 mm) was purchased from Nature Works (Product name: Ingeo™ biopolymer and Product code: 4043D, Naarden, The Netherlands), with a processing temperature set in the range 200–218 °C. SolidWorks was used to design PLA samples according to ASTM D638 standard. The developed STL files were transmitted to the Ultimaker Cura 4.11.0 for slicing the specimen to custom parameters of infill density, infill geometry, and infill line multiplier. This programme managed and monitored print progress, as well as queueing prints and various material allocations. The specimens were printed using a commercial Ultimaker S3 3D (Geldermalsen, The Netherlands) printer using a nozzle with a diameter of 0.4 mm. Custom printing settings were utilised for printing to give the printing programme more control, allowing the printer to define printing parameter settings, such as line multiplier, pattern, and infill ratios. The printing speed was set at 70 mm/s, and the build plate temperature was set at 60 °C [35]. Batches 1–3 were printed to determine the effect of the infill pattern (Figure 1).

**Figure 2:**
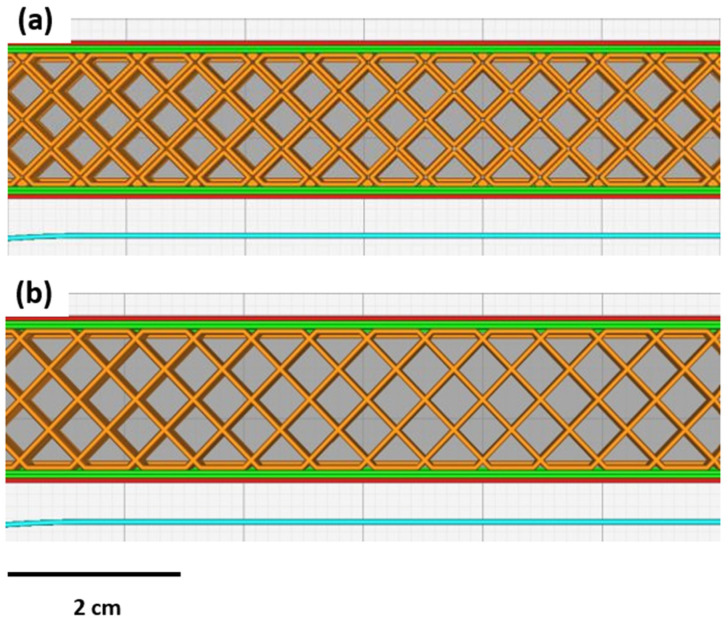
(**a**) Infill line multiplier 2; (**b**) infill line multiplier.

Batch 4 was printed to determine the effect of infill line multiplier on the mechanical behaviour of the 3D-printed PLA. The infill patterns for the 3D-printed samples are shown in Figure 2.

From both batches, a total of 33 samples were printed. Table 1 demonstrates various printing parameters employed during the printing of samples.

Batch 4 differed in the infill line multiplier of specimens. The infill line multiplier 1 had an infill density of 50%, while the infill line multiplier 2 had an infill density of 25%. These combinations were selected to maintain same weight (5.5 g) for the specimens and investigate the effect of the infill line multiplier on mechanical properties. The printed samples were left to cool on the bed and removed once they are at room temperature to avoid any damage to printed structure. The specimens were then weighed to determine their mass, and their dimensions were measured using digital calipers.

For mechanical testing, a Universal Testing System (3382A Floor Model, Instron, Buckinghamshire, UK), which was fitted with a load cell with a capacity of 100 kN, was used. The maximum speed of this machine is 508 mm/min (20 in/min), guaranteeing the precision of received data. Tensile testing was carried out at a speed of 4 mm/min. Bluehill Universal with a data capture rate of 500 Hz was used to gather and present the results. The UTS, Young’s Modulus, and elongation at break for each specimen were determined through analysing the stress–strain curves obtained from the tensile tests. An average value was taken for the infill densities and infill patterns. The mean error obtained for stress was 0.017711 MPa, while for strain the mean error was 0.000277.

## 3. Results and Discussion

**Figure 3:**
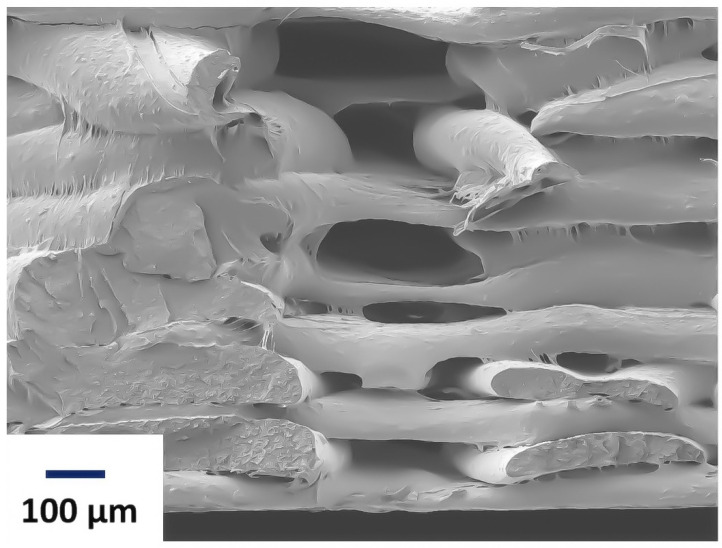
Scanning Electron Micrograph of PLA with hexagonal infill pattern at 75% infill density.

Figure 3 shows a Scanning Electron Microscope (SEM) image of the fractured surface of 3D-printed PLA samples with a hexagonal infill pattern and a 75% infill density. The fracture appears to exhibit irregularities and jagged edges, indicating a brittle or partially brittle fracture mode. Although not explicitly visible in the SEM image, the 75% infill density suggests that the internal structure of the printed object likely comprises a lattice-like pattern or a series of interconnected struts, which is characteristic of 3D-printed objects [36]. These layers represent individual printing passes or deposition lines that build up the final object. The bonding and interaction between these layers impact the overall strength and integrity of the printed part. This structure provides mechanical support and stability to the printed part while reducing overall weight.

### 3.1. Effect of Infill Pattern

Figure 3 displays a comparative analysis of various infill patterns as a function of the applied stress and strain. The amount of material used also increases with increasing infill density, and objects with 25%, 50%, and 75% infill density weigh 3.9 g, 5.9 g, and 8 g, respectively. All specimens with grid, triangular, and hexagonal infill patterns initially exhibited linear elastic behavior, which transitioned to non-linear viscoelastic behavior. After reaching the initial peak, the deformation behavior of specimens with hexagonal and triangular infill patterns differed. The 3D-printed PLA samples exhibited strain softening, and the strain hardening response was very limited. This characteristic is associated with the brittle behaviour of the material [37,38]. The UTS of the samples decreases as the infill density decreases; however, the relationship is not linear. Different infill patterns have different effects on the stress–strain curve. For example, the 75% triangular pattern has a significantly lower area under the curve compared to 75% hexagonal pattern [13], depicting lower toughness values. One of the possible explanations for increased toughness and higher UTS values could be that the hexagonal infill pattern offers better structural integrity through its symmetry and interconnectedness, distributing the load uniformly. It has more material bridges for uniform load distribution, enhancing support and reinforcement. Furthermore, increased interlocking between layers prevents separation, increasing strength. On the other hand, the triangular infill pattern does not distribute the force evenly throughout the object; hence, the print is likely to fail at lower loading values.

However, the relationship between the amount of material and the toughness is not straightforward, as the type of infill pattern also has an impact on the resistance to deformation. Moreover, there is a proportional reduction in the UTS values with decreasing infill density. Hexagonal pattern exhibited the highest UTS of all the patterns. Therefore, it can be concluded that each pattern has a distinct impact on mechanical strength. Interestingly, the strain behaviour for all of the samples exhibited similar values for all infill patterns. However, the stress value varies for different infill patterns. This result means that different infill patterns can withstand different amounts of force before they fail. The fact that the strain at failure is the same for different infill densities of 3D-printed samples suggests that infill density does not significantly affect the ultimate deformation capacity of printed objects. This result indicates that the infill density does not directly affect the material’s ability to withstand deformation before failure.

There could be several reasons for this observation. One of the possible explanations for this behaviour lies in the fact that different infill patterns affect the way that the force is distributed throughout the object. Moreover, the strain at failure may be primarily governed via the mechanical properties of the material itself, rather than the internal structure created using the infill pattern. Infill density primarily impacts factors such as weight, strength, and stiffness; however, it may have a limited effect on the material’s intrinsic ability to deform under stress.

**Figure 4:**
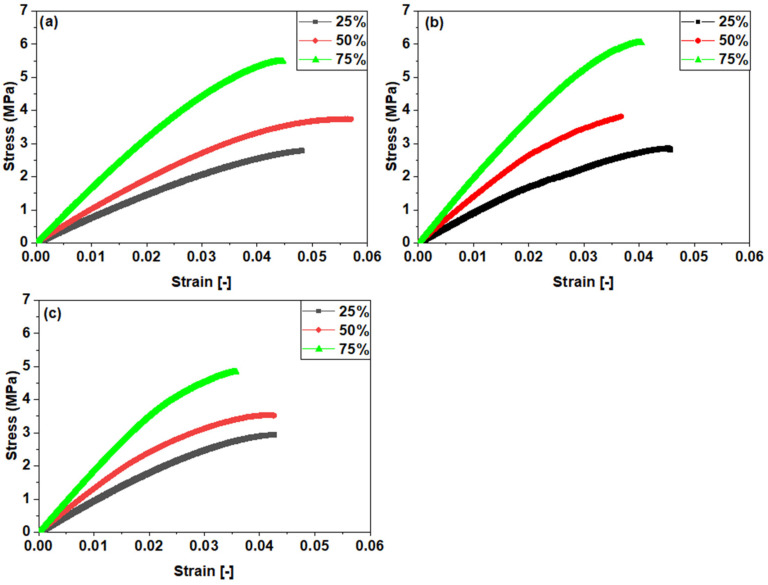
Effect of infill density as a function of infill patterns: (**a**) grid pattern; (**b**) hexagonal pattern; (**c**) triangular pattern.

**Figure 5:**
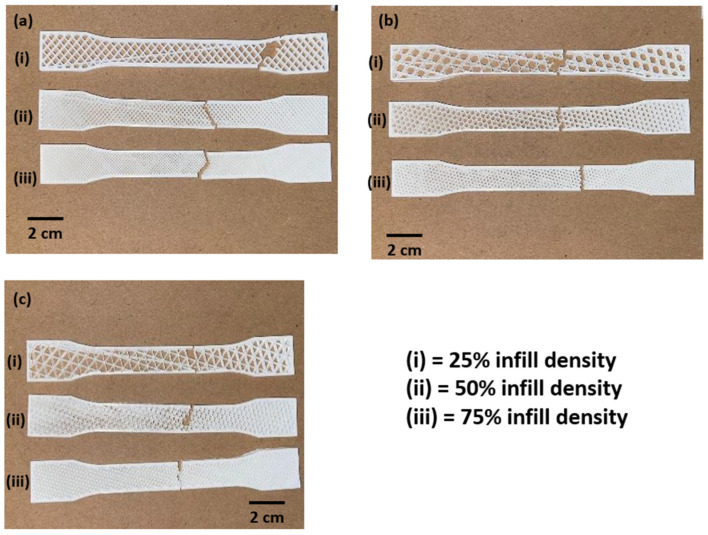
Failure of samples after tensile test: (**a**) grid pattern; (**b**) hexagonal pattern; (**c**) triangular pattern.

Following first strut fracture, the displacement curves were in the displacement range of 2.24 to 3.02, as seen through cracking and tiredness. Random samples are captured to demonstrate the fatigue line and the precise location where the pieces fractured. Each sample was examined individually to assess the strength of each specimen under tensile stress, as shown in Figure 4. Three specimens were evaluated at each infill pattern, and the resulting loads were recorded. The results showed that the impact strength decreased as the infill density decreased. The lower infill density of printed samples resulted in fewer printing layers, larger gaps between them, and higher porosity in the mesostructure. These factors decreased the interlayer bonding strength and increased the stress-induced cracking as shown in Figure 5. The specimen with 25% infill density appeared to have a different fracture behaviour than the rest of the samples. The fracture was at a certain angle, which propagated throughout the sample. This behaviour was observed in all the samples that were printed using grid infill pattern. Although the grid and triangular pattern are physically close in likeness, the fracture behaviour was different. It appeared that the resisting regions, in the case of the grid pattern, were more responsible for causing the crack to propagate irregularly within the structure, thus giving an irregular fracture pattern [2].

As a result, Table 2 shows the average maximum tensile load for flat samples, demonstrating the range of weights required to break specimens The maximum loads for a hexagonal design were roughly 1081N, 696N, and 473N for infill ratios of 75%, 50%, and 25%, respectively.

### 3.2. Effect of Infill Density

**Figure 6:**
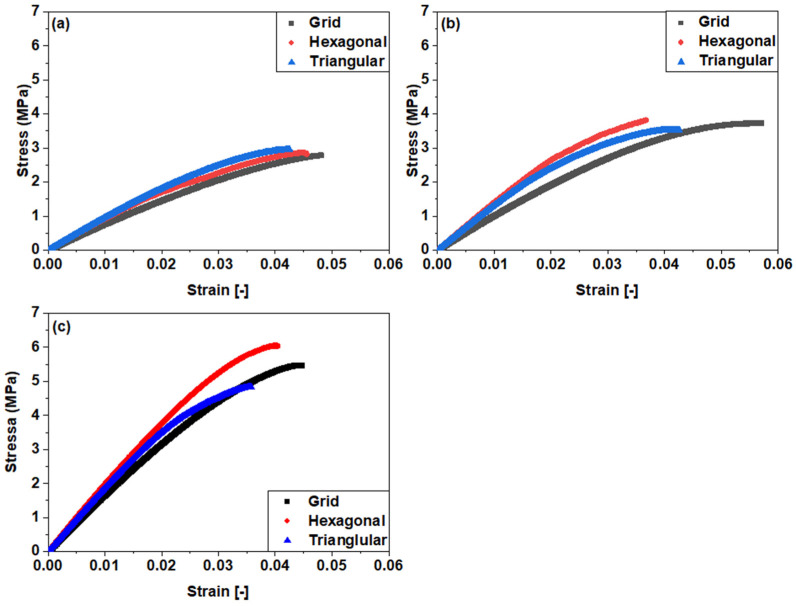
Effect of infill patterns as a function of infill densities: (**a**): 25%; (**b**): 50%; (**c**): 75%.

Infill density is another crucial factor that impacts the mechanical performance of 3D-printed products. Figure 6 illustrates the relationship between various infill patterns and infill densities on stress and strain behaviour of 3D-printed samples. The results confirm the substantial influence of infill densities (25%, 50%, and 75%) on the tensile strengths of the tested samples. Notably, the results demonstrate a nearly linear correlation, indicating that increasing the infill density leads to higher tensile strength [39]. At lower infill densities, the stress–strain curves showed a more gradual increase in stress until failure, indicating more ductile behaviour. In contrast, at higher infill densities, the stress–strain curves showed a more sudden increase in stress until failure, indicating more brittle behaviour. The alteration in the behaviour can be attributed to the transformation in the internal configuration of the printed component with the increase in infill density [35]. The hexagonal infill pattern demonstrated a high tensile strength at all infill densities, with the highest being at 75% infill density. This pattern’s ability to distribute and transfer stress more effectively within the printed structure may contribute to its superior mechanical performance. On the other hand, the grid pattern consistently exhibits the lowest UTS across all infill densities studied. This outcome suggests that the grid pattern may not offer optimal load-bearing capabilities or efficient stress distribution, resulting in reduced overall tensile strength.

**Figure 7:**
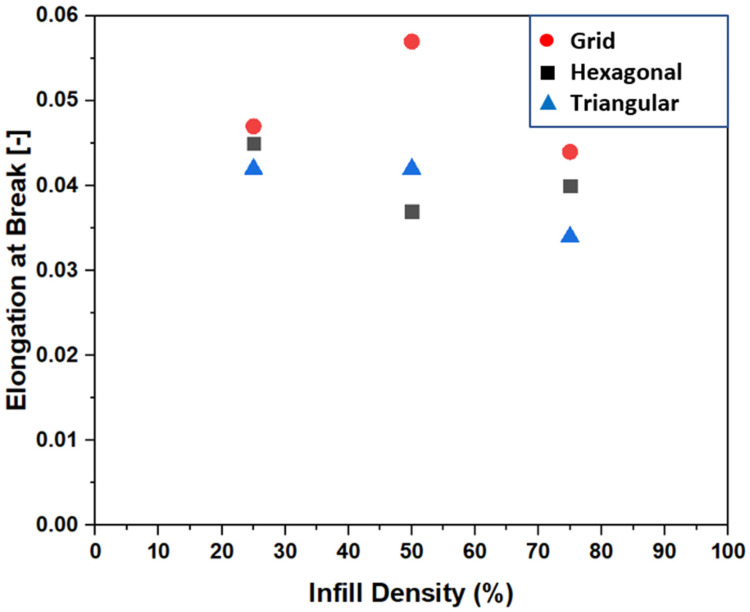
Elongation at break of infill patterns as a function of infill densities.

Figure 7 shows the relationship between infill density and elongation at break. The graph shows a consistent decreasing trend in elongation at break with increasing infill density. The elongation at break shows a very minimal decrease, averaging around 0.04. This result suggests that while the infill density has a significant impact on UTS, elongation at break remains nearly constant. Furthermore, it was observed that higher infill densities generally result in reduced ductility, decreasing the ability of a 3D-printed specimen to deform plastically and sustain elongation. While the strain at break remains relatively constant, the decrease in ductility highlights the trade-off between strength and deformability when choosing higher infill densities. This trade-off is an important consideration in applications where both high strength and adequate ductility are required.

**Figure 8:**
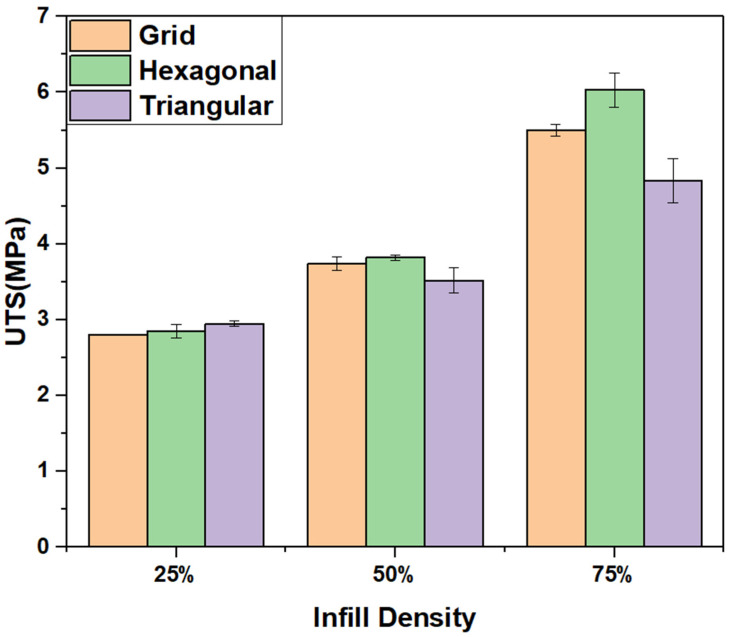
Effect of UTS for various infill patterns as a function of infill densities for a 1-line multiplier.

A clear trend of increasing strength and stiffness with increasing infill density was observed for all infill patterns, as shown in Figure 8. The 3D-printed PLA specimens’ ultimate tensile strength and Young’s Modulus demonstrated a significant rise when the infill density was enhanced from 25% to 75%. Specifically, for the hollow structures printed using hexagonal infill patterns, the ultimate tensile strength rose from 2.85 MPa (25% infill density) to 6.03 MPa (75% infill density). The improved mechanical properties are attributed to the more compact internal structure that results from having a higher infill density [2,13]. However, it is important to note that increasing the infill density can also increase the printing time and material usage, which can increase the overall cost of production. Therefore, the desired mechanical properties must be balanced against the cost of production when choosing the infill density for 3D printing of PLA parts.

Table 3 shows values of Young’s Modulus in relation to different infill patterns and infill percentages. Young’s Modulus is a fundamental property that characterises the stiffness or elasticity of a material. The decrease in Young’s Modulus correlated with the infill percentage decreases within each infill pattern can be attributed to the presence of voids or empty spaces within the structure. As the infill percentage decreases, the amount of material filling the structure decreases, leading to larger voids or air gaps. These voids act as stress concentrators and reduce the overall stiffness of the material. Consequently, materials with lower infill percentages exhibit more flexibility and less resistance to deformation.

Secondly, the variation in Young’s Modulus values among different infill patterns at the same infill percentage suggests that the arrangement of the material within the structure plays a significant role in determining the stiffness of the material. Each infill pattern has a unique geometric configuration, and these geometric differences affect how the applied stress is distributed throughout the material. For example, the hexagonal pattern 0.89, with its closely packed hexagonal cells, provides a more efficient load-bearing structure, resulting in a higher Young’s Modulus compared to the grid or triangular patterns. Interestingly, the results of introducing a 2-line multiplier to the 25% infill density in the hexagonal pattern showed an increase in the Young’s Modulus, suggesting that the multiplier has an impact on the stiffness of the material. These findings underscore the importance of careful selection of the infill pattern and infill percentage in controlling the mechanical properties of materials. Engineers and designers can leverage this knowledge to tailor materials for specific applications. For instance, in structural engineering, a material with a higher Young’s Modulus may be preferred to ensure rigidity and load-bearing capacity, while in applications where flexibility is desirable, materials with lower Young’s Modulus may be more suitable.

### 3.3. Effect of an Infill Line Multiplier

**Figure 9:**
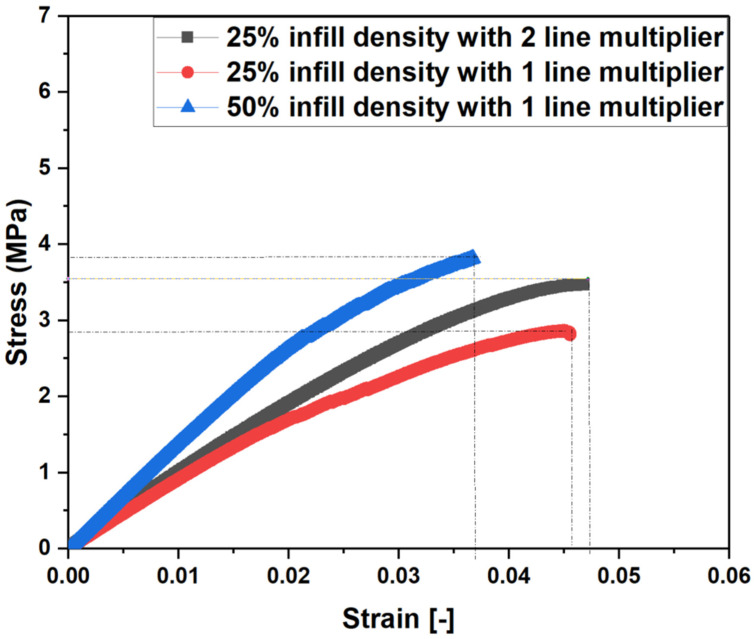
Effect of infill line multiplier as a function of infill densities.

To investigate the influence of infill line multipliers on mechanical properties, a hexagonal infill pattern was chosen with a one- and two-line infill multiplier. To ensure consistency, specimens weighing 5.5 g were targeted for both cases, as it was found that infill percentages of 25% with a two-line multiplier and 50% with a one line multiplier would give a wight of 5.5 g. It was observed that an increase in line multiplier leads to better mechanical properties, as shown in Figure 9. A 25% infill density with two-line multiplier showed comparable UTS to a 50% infill density with a two-line multiplier. This combination exhibited a UTS value of 3.57 MPa, which is very close to the value of samples printed at 50% infill density, which was 3.83 MPa. Interestingly, the elongation at break stayed the same, suggesting that the printed specimen can sustain higher load before breaking. This result is attributed to the fact that wider lines have better inter-layer bonding, which improves the strength of the product while keeping the hollow structure intact. However, there is a limit to the increase in line multiplier, beyond which the mechanical properties start to deteriorate [40]. This fact is because wider lines may lead to over-extrusion, which can cause deformation, warping, or voids in the printed object [10]. Moreover, excessive over-extrusion can reduce the accuracy and resolution of the printed product, which may be undesirable in some applications.

Table 4 shows the UTS and weight of samples printed with a hexagonal infill pattern, with varying infill densities. It can clearly be seen that the Strength-to-Weight Ratios of the samples printed with 25% infill density are comparable to the sample printed with 50% infill density. Using a line multiplier in 3D printing for hollow structures offers increased structural integrity by increasing the density of internal support lines without compromising the porosity. Moreover, the use of line multipliers also reduces print times compared to uniformly increasing infill density.

It is important to note that the effect of the line multiplier on the mechanical properties of 3D printed objects can vary depending on the material used, the printing parameters, and the design of the object. Therefore, it is important to optimise the line multiplier for each application to achieve the desired mechanical properties while maintaining the printing quality.

## 4. Conclusions

In conclusion, this paper investigated the effects of infill line multiplier and different infill patterns (Hexagonal, Grid, and Triangle) on the mechanical properties of Fused Filament Fabrication (FFF)-printed hollow structures using thermoplastic poly lactic acid (PLA) as the printing material. All of the samples demonstrated increasing UTS with increasing infill densities. However, the strain at failure stayed nearly the same for all of the samples. The results revealed that the hexagonal infill pattern consistently exhibited the highest Ultimate Tensile Strength (UTS) of 1.86 MPa across all infill densities, outperforming the other patterns. The enhanced performance of the hexagonal infill pattern was due to the increased interlocking and uniform load distribution. The findings highlight the significant influence of the combined effect of infill density and line multiplier on the weight, strength, and stiffness of the printed objects. A line multiplier of 2 combined with a 25% infill density achieved a UTS value of 3.57 MPa, which is comparable to samples printed at 50% infill density with 1 line multiplier. These findings suggest that for hollow structures, infill line multipliers have higher mechanical strength compared to corresponding samples printed with 1-line multipliers.

Infill patterns and line multiplier comparison in fused filament fabrication play a critical role in developing lightweight and resilient hollow section products. The selection of an appropriate infill pattern and line multiplier directly impacts the stress and strain values of the final product. The UTS values of the product are also significantly influenced by the infill pattern and line multiplier selection. Therefore, it is important to carefully evaluate the infill patterns and line multiplier options available in fused filament fabrication to produce a product with the desired mechanical properties. Future research in this area could explore additional factors that impact the strength and durability of hollow-section products, such as the effect of environmental conditions and post-processing techniques.

## Figures and Tables

**Table 1:** Geometric printing parameters.

Batch No	Pattern	Infill Density %	No of Specimens
Infill geometry and infill pattern as main criteria			
1.	Hexagonal	25, 50 and 75	3 per each density
2.	Grid	25, 50 and 75	3 per each density
3.	Triangular	25, 50 and 75	3 per each density
Infill line multiplier as main criteria			
4.	Hexagonal	25 and 50	3 per each density

**Table 2:** Mechanical properties’ values for different infill densities.

Infill Pattern	Infill Pattern	Maximum Load	Maximum Displacement at Failure
**Grid**	75%	895N	2.24 mm
	50%	583N	2.94 mm
	25%	455N	2.56 mm
**Hexagonal**	75%	1081N	2.04 mm
	50%	696N	2.12 mm
	25%	473N	2.27 mm
**Triangular**	75%	764N	1.81 mm
	50%	540N	2.15 mm
	25%	467N	2.13 mm

**Table 3:** Young’s Modulus values of PLA for different infill densities.

Infill Pattern	Infill Pattern	Young’s Modulus (MPa)	Standard Deviation
**Hexagonal**	75%	1.86	±0.12
	50%	1.35	±0.02
	25%	0.89	±0.05
	25% with a 2-line multiplier	0.93	±0.11
**Grid**	75%	1.63	±0.08
	50%	0.96	±0.09
	25%	0.73	±0.01
**Triangular**	75%	1.79	±0.02
	50%	1.3	±0.08
	25%	0.90	±0.02

**Table 4:** Strength-to-Weight Ratio of PLA for different infill densities.

Infill Pattern	Infill Pattern	Ultimate Tensile Strength (MPa)	Weight (g)
**Hexagonal**	75%	6.03	8.25
	50%	3.82	5.5
	25%	2.85	2.8
	25% with 2-line multiplier	3.52	5.5

## Data Availability

Data can be provided upon request.
